# Expression of RFC/SLC19A1 is Associated with Tumor Type in Bladder Cancer Patients

**DOI:** 10.1371/journal.pone.0021820

**Published:** 2011-07-08

**Authors:** Alyaa M. Abdel-Haleem, Maha I. El-Zeiry, Laila G. Mahran, Khaled Abou-Aisha, Mona H. Rady, Jan Rohde, Marwa Mostageer, Hilde Spahn-Langguth

**Affiliations:** 1 Department of Pharmacology and Toxicology, German University in Cairo, Cairo, Egypt; 2 Department of Pharmaceutical Chemistry, Institute of Pharmaceutical Sciences, University of Graz, Graz, Austria; Howard University, United States of America

## Abstract

Urinary bladder cancer (UBC) ranks ninth in worldwide cancer. In Egypt, the pattern of bladder cancer is unique in that both the transitional and squamous cell types prevail. Despite much research on the topic, it is still difficult to predict tumor progression, optimal therapy and clinical outcome. The reduced folate carrier (RFC/SLC19A1) is the major transport system for folates in mammalian cells and tissues. RFC is also the primary means of cellular uptake for antifolate cancer chemotherapeutic drugs, however, membrane transport of antifolates by RFC is considered as limiting to antitumor activity. The purpose of this study was to compare the mRNA expression level of RFC/SLC19A1 in urothelial and non-urothelial variants of bladder carcinomas. Quantification of RFC mRNA in the mucosa of 41 untreated bladder cancer patients was performed using RT-qPCR. RFC mRNA steady-state levels were ∼9-fold higher (N = 39; P<0.0001) in bladder tumor specimens relative to normal bladder mRNA. RFC upregulation was strongly correlated with tumor type (urothelial *vs.* non-urothelial; p<0.05) where median RFC mRNA expression was significantly (p<0.05) higher in the urothelial (∼14-fold) compared to the non-urothelial (∼4-fold) variant. This may account for the variation in response to antifolate-containing regimens used in the treatment of either type. RFC mRNA levels were not associated with tumor grade (I, II and III) or stage (muscle-invasive *vs.* non-muscle invasive) implying that RFC cannot be used for prognostic purposes in bladder carcinomas and its increased expression is an early event in human bladder tumors pathogenesis. Further, RFC can be considered as a potential marker for predicting response to antifolate chemotherapy in urothelial carcinomas.

## Introduction

20–60% of the cancer incidence worldwide has been associated with nutritional factors [1]. Among the most extensively studied nutrients involved in cancer etiology is the vitamin folic acid. Folates are essential for life, and folate deficiency contributes to a host of health problems including cardiovascular disease, fetal abnormalities, neurological disorders, and cancer [1], [2].

Cancer of the bladder is the ninth most common cancer worldwide. The majority (77%) of bladder tumors occur in men. It is the seventh most common malignancy in men and 17th in women [3]. The spectrum of bladder tumors is broad and includes transitional cell carcinoma (TCC), squamous cell carcinoma (SCC), adenocarcinoma and small cell carcinoma. TCC is by far the most prevalent and best studied. In the United States, 90% of bladder tumors are of the urothelial variant that evolves from transitional cells. Conversely, in Egypt, the most common histopathological type of bladder cancer used to be SCC [4], constituting from 59% to 81% of reported bladder cancers between 1960 and 1980 [4], [5]. Contrary to the leading etiology of smoking and occupational exposures in Western countries, chronic bladder infection with *Schistosoma haematobium*, has been the most important risk factor for bladder cancer in Egypt [5]. However, recent studies [5], [6] reported that SCC in Egypt appears to have decreased from 78% in 1980 to 27% in 2005.

Biopsy samples of the bladder tumor lesions can be easily obtained by trans-urethral resection (TUR) prior to treatment. Despite much research on the topic, it is still difficult to predict tumor progression, optimal therapy and clinical outcome [7]. Tumor staging (TNM system) is considered to be the best prognostic marker; however, failure to accurately predict progression or recurrence is often experienced [8]. In addition, treatment failure is a major area in which translational bladder cancer research could benefit patients. For instance, the MVAC regimen (an antifolate-containing regimen) exhibits only limited antitumor activity against non-transitional cell cancer while response rates up to ∼70% were reported in TCC in the same study [9]. If genetic discrepancies between the two variants of bladder carcinomas affect their progression and/or response to chemotherapy, then the question is; which genetic marker(s) is (are) the most critical ones?

The ubiquitously expressed reduced folate carrier (RFC) is considered the major transport route for folates at physiological cofactor concentrations even when multiple uptake systems are present [1], [10], [11]. Loss of RFC expression or function portends potentially profound physiological and developmental consequences [1], [12]. RFC is also a major transporter of antifolate drugs used for cancer chemotherapy such as methotrexate (MTX), pemetrexed, and raltitrexed. The effectiveness of chemotherapy with these agents is closely linked to levels and activity of RFC in both tumors and normal tissues [13], [14]. MTX, for instance, continues to be an important component of the chemotherapeutic armamentarium for a variety of malignancies including bladder cancer [15], [16]. Pemetrexed has also been recently considered for use in treatment of bladder cancer [16]. Membrane transport of these antifolates is critical to antitumor activity since this provides sufficient (unbound) intracellular drug to sustain maximal inhibition of enzyme targets (e.g., DHFR; Dihydrofolate reductase) and for synthesis of polyglutamate derivatives required for high affinity binding to intracellular enzymes [13]. The reduced folate carrier gene expression can be reliably studied at the mRNA level, and based on a number of studies [13], [14], [17], [18], [19], [20], [21] the expression is likely to reflect the activity of the RFC in both tumors and normal tissues. Hence, inspecting discrepancies in RFC expression among the different types of bladder cancers may pave the way for possible improvement of rationally based antifolate-containing chemotherapy.

The present study was, thus, undertaken to investigate mRNA expression and potential clinical relevance (i.e. associations with tumor stage, grade and/or histological type) of the major folate transporter, RFC, in a group of human urinary bladder tumors of different histological types.

## Materials and Methods

### Ethical statement

An initial number of 61 bladder cancer patients were included in this study. All patients' data were analysed anonymously and patients' consent had been obtained to include excised tissue for the current study via the pathology department at the National Cancer Institute (NCI), Cairo, Egypt. All research protocols were approved by the institutional review boards of the NCI and the Research Ethics Committee of the German University in Cairo.

### Patients

Sixty one tumor biopsies were obtained by TUR at the NCI during the period from March 2009 to January 2010. 17 specimens were later excluded from the study during the RNA quality check step (see below). For the remaining 44 specimens, tumor stage and grade were assigned according to the TNM [4] by the pathology unit at the NCI.. Patients included in this study (n = 44; 31 males , 11 females and 2 unidentified; age range: 31 to 79) were allocated into two main groups depending on tumor histological type: urothelial tumors' group; TCC and TCC with squamous metaplasia; and non-urothelial tumors' group; SCC and adenocarcinoma. At the time of inclusion, none of the patients had received treatment in any form. Clinical characteristics of the bladder cancer patients included in this study are listed in Table S1.

### Total RNA extraction and reverse transcription

Excised tumor biopsies used for mRNA analysis were immediately placed in RNA*later*® RNA Stabilization Reagent (Qiagen, Germany), kept at −4°C overnight then stored at −20°C. Total RNA was extracted from frozen biopsies (∼30 mg) using Absolutely RNA mini-prep kit (Stratagene, Germany) according to the manufacturers' instructions. Isolated RNA integrity was electrophoretically verified by ethidium bromide staining and by A_260_/A_280_ absorption ratio (range 1.8–2.0) (A260 nm = 1 corresponds to 40 µg/ml RNA). A 2∶1 intensity ratio between 28S and 18S rRNA was considered a benchmark for intact RNA (Figure S1). 17 tumor specimens were excluded from the study based on their RNA integrity profile (Figure S1). 5 µg total RNA was reverse transcribed with Sprint™ RT Complete Products kit (Clonetech, Germany) in a volume of 20 µl according to the manufacturers' instructions.

### RT-qPCR quantification of RFC mRNA

The RT-qPCR protocol followed in this study was structured around the Minimum Information for Publication of Quantitative Real-Time PCR Experiments (MIQE) guidelines, published by Bustin *et al.* [22]. RFC mRNA was quantified by RT-qPCR on Mx3005P™ instrument (Stratagene, USA). Transcripts of β-actin were quantified as an endogenous RNA control [7], [23] to eliminate variations in the amount and/or quality of total RNA added to each reaction mix and each sample was normalised on the basis of its β-actin mRNA content. All the quantifications in this study are presented as the ratio between the target gene and β-actin. Primer sequences, annealing temperature and PCR products size for the target gene RFC and the reference gene β-actin are presented in Table 1. The specificity of each primer pair was confirmed by melting curve analysis (Figure S2) which resulted in single product specific melting temperatures.

**Table 1:** Characterisitcs of gene-specific real-time PCR assays.

Gene/Accession No.	Mer	Amplicon	Efficiency(%)	Conc.	Resources
**SLC19A1 [GenBank:NM_194255.1]**					Primer Express®
FWD: CCT CGT GTG CTA CCT TTG CTT	21	125	105.8	100 nM	
REV: TGA TCT CGT TCG TGA CCT GCT	21			100 nM	
**ACTB [GenBank:NM_001101.1]**					Ohl *et al.* [23]
FWD: AGC CTC GCC TTT GCC GA	17	174	93	300 nM	
REV: CTG GTG CCT GGG GCG	15			300 nM	

Each primer pair was evaluated for secondary structures and SNPs by the *in silico* evaluation tool at RTPrimerDB (http://medgen.ugent.be/rtprimerdb). Highly purified salt-free primer for RFC and β-actin (Table 1) were generated commercially (Metabion GmbH, Germany) and optimised to a two-step (combined annealing/extension temperature) PCR protocol. A pool of 3 bladder cancer cDNA samples was used for assay optimization and amplification efficiency evaluation (Table 1) using “the standard curve” method. A standard curve for each gene was then generated automatically by the Mx3005P™ software where log the target concentration was plotted against the corresponding quantification cycle (Cq) value [22]. Conditions for all PCRs were optimised with regard to forward and reverse primers concentrations and various annealing temperatures (55–66°C). Real-time PCR mastermix of the following reaction components was prepared to the indicated end-concentration: 12.5 µl Power SYBR® Green PCR Master Mix (Applied Biosystems, UK), 0.2 µl forward primer RFC (100 nM), 0.2 µl reverse primer RFC (100 nM) and 11.1 µl nuclease-free water for the target gene; and 12.5 µl Power SYBR® Green PCR Master Mix, 0.75 µl forward primer β-actin (300 nM), 0.75 µl reverse primer β-actin (300 nM) and 10 µl nuclease-free water for the reference gene. 24 µl of the prepared mastermix were pipetted per well in a 96-well plate. 1 µl cDNA template was then added to each reaction well. Applications of total cDNA input were performed in duplicates. The 96-well plate was sealed by a PCR optical adhesive film (Eppendorf AG, Germany). The following optimized thermal profile was used: denaturation program (95°C for 10 min), amplification and quantification program repeated 40 times (95°C for 15 s, 60°C for 1 min, with a single fluorescence measurement), melting curve program (55–95°C with a heating rate of 0.1°C per second and a continuous fluorescence measurement). The fluorescence data were collected and the mRNA quantified with Mx3005P™ software version 4.1. Cq values were automatically calculated according to the “fit point method”; where Cq is defined as fractional number of cycles at which the PCR kinetic curve reaches a program-defined threshold amount of fluorescence.

The fold change in expression of β-actin was estimated according to the method of Schmittgen and Livak [24]. As shown in Table 2, the variation in β-actin expression was minimal among the urothelial and non-urothelial groups.

**Table 2:** Fold change in the reference versus target genes expression levels among the different histological subtypes of bladder cancer.

Compared Groups	β-actin fold difference	RFC fold difference
Urothelial: non-urothelial	1.49	7.93

### Statistical Analysis

The data were tested for normality using the Shapiro-Wilk normality test. Nonparametric tests were decided accordingly. P-values less than 0.05 were considered to be significant. The Mann–Whitney U-test and Kruskal–Wallis test were used to correlate the expression of the RFC with tumor stage, grade and type. The software *Graph Pad Prism* (version 5) was used for statistical analysis.

## Results

### mRNA expression of RFC

The mRNA expression of the folate transporter RFC was quantified in 41 bladder cancer specimens, three specimens with no evidence of malignancy obtained from bladder mucosa adjacent to cancerous tissue, and a commercially available reference bladder RNA pooled from 26 male/female Caucasians; aged 22–70 (Clonetech, Germany). The latter will be referred to as the calibrator in the following sections.

RFC mRNA expression was detected in all bladder specimens used in this study. The steady-state expression levels of RFC mRNA were significantly (*P*<0.0001) higher in the bladder cancer specimens by ∼9-fold (8.661, range 0.98–50, N = 39) than in the calibrator.

### Clinical *vs.* hisotpathological parameters

**Figure 1:**
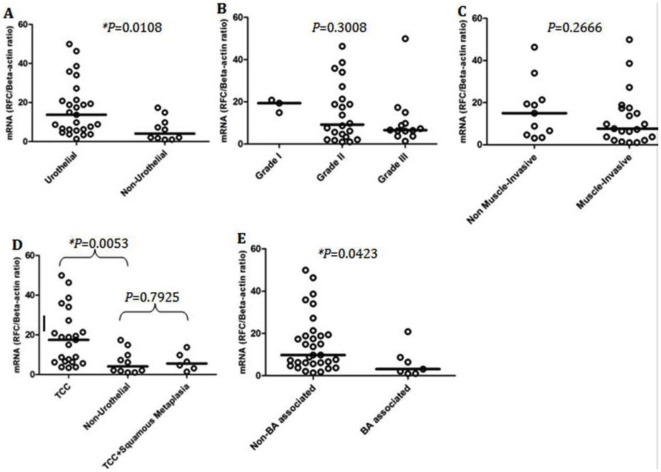
mRNA expression of RFC in 39 human biopsy samples relative to the calibrator (A–E). Solid horizontal lines demonstrate the median of each individual group. All data are shown as the ratio between the target gene and beta-actin. *P-value, non-parametric, Mann-Whitney or Kruskal-Wallis tests. BA, Bilharizal-associated.

A multivariate analysis was done to determine the most significant factors with which RFC mRNA expression was associated. The correlation between RFC mRNA levels and the tested clinicopathological parameters of bladder cancer are summarised in Table 3. RFC relative expression level was significantly (*P*<0.05) higher in urothelial *vs.* non-urothelial bladder carcinomas where RFC mRNA was increased by ∼14-fold (13.27; n = 27) in urothelial tumors in comparison to 4-fold (4.083; n = 10) increase only in the non-urothelial variants (Figure 1A). The median expression level of RFC in the TCC specimens was 17.48; n = 23 in comparison to 5.538; n = 6 in specimens classified as TCC with squamous metaplasia or mixed transitional and squamous cell carcinomas. However, no statistically significant difference was found between RFC mRNA expression in bladder cancer specimens classified as pure TCC *vs.* those having TCC with squamous cell metaplasia (Figure 1D). This shows that RFC mRNA expression in non-urothelial tumors is much lower than in their urothelial counterparts and the squamous component is predominantly associated with reduced RFC mRNA expression. The expression level of RFC in bladder cancer specimens containing bilharzial ova was compared to those with no evidence of bilharzial infection where RFC mRNA was significantly (*P*<0.05) decreased in bilharzial-associated (BA) bladder cancer specimens (3.13; n = 7) in comparison with the non-bilharzial-associated group (9.76; n = 32) (Figure 1E).

**Table 3:** Correlation between RFC mRNA levels and clinicopathological parameters of bladder cancer.

	Tumor type^a^	Grade^b^	Stage^c^	Bilharziasis^d^
Median RFC mRNA expression relative to the calibrator	P<0.05^e^	ns	ns	P<0.05

a Urothelial *vs.* non-urothelial.

b I *vs.* II *vs.* III.

c Muscle-invasive *vs.* non-muscle invasive.

d Bilharzial-associated *vs.* non-bilharizal-associated bladder tumor.

e *P*-values (Mann-Whitney and Kurskal-Wallis tests); NS, not significant.

We examined the correlation between levels of expression of RFC mRNA and markers of tumor aggressiveness (namely; grade and stage). The expression of RFC mRNA didn't correlate with tumor grade (Figure 1B) where there was no statistically significant difference (*P* = 0.3008) detected between grades I, II, or III. Further, RFC expression was not statistically altered (*P* = 0.2666) between muscle-invasive and non-muscle invasive specimens (Figure 1C). Notably, all SCC specimens (n = 8) were classified as muscle-invasive tumors, which points to the more aggressive nature of SCC and non-urothelial carcinomas. In addition, no statistically significant difference (*P* = 0.2162) was found between the ages of patients with urothelial carcinomas (mean = 60.42) *vs.* those of the non-urothelial group (mean = 52.88).

## Discussion

Since mammalian cells cannot synthesize folates *de novo*, tightly regulated cellular uptake processes have evolved to sustain sufficient levels of intracellular folates [25], [26]. In context of malignancy, the major folate transporter system, RFC, increased expression is consistent with the need to increase cellular retention of folates to support the requirements of the rapidly dividing cancer cells for DNA synthesis and replication. In the present study, our findings in human bladder cancer biopsies were in accordance with this notion where RFC mRNA expression was significantly increased by ∼14-fold in urothelial bladder carcinomas (TCC and TCC with squamous metaplasia) and by ∼4-fold in the non-urothelial variants (SCC & Adenocarcinoma). Altered expression of RFC has been also reported in leukemias, particularly B-precursor ALL [11], [26], [27], osteosarcoma [17], colorectal cancer and primary central nervous system lymphomas [1] at both the transcript and protein levels.

The discrepancy in RFC mRNA expression between urothelial and non-urothelial bladder tumors, reported in the current study, could be attributed to upstream genetic events that take place in each type, which may affect transcriptional regulation of the transporter. Both ubiquitous and tissue-specific TFs that transactivate or repress transcription in response to requirements for folate cofactors control the net levels of RFC transcripts in tissues [1], [18]. Literature and pathway mining using the *LitInspector* software [28] showed that RFC (SLC19A1) has potential interactions with CREB1 (CAMP responsive element binding protein-1). CRE/ AP-1-like consensus sequence is one of the two major sites of regulation of the RFC basal promoters [18]. CREB, which binds to the CRE/AP-1, is among the TFs activated by the ERKs (MAPK), which are components of the RTK-RAS signaling pathway. Accruing evidence indicates that key components of this signaling pathway are frequently activated in human urothelial carcinomas [29], but not in the non-urothelial types [30], [31]. In human fibrosarcoma cells, CREB-1 and c-Jun were the cell-specific transcription factors involved in CRE binding in the RFC promoter. Similar findings were reported for CREB-1 and ATF-1 in human hepatocellular carcinoma cells and human leukemia cells [18], [32]. Furthermore, two thirds of the antifolate-resistant cell lines displayed a marked decrease in transcription factor binding to a single CRE site present in the minimal promoter A of the RFC gene [33]. Hence, there is increasing evidence to suggest that binding of CREB-1 to CRE in the RFC promoter is an important contributor to the induction of RFC gene expression in cancer cell lines and malignant tumors [18], [32], [33]. Therefore, we hypothesize that the constitutive activation of the RTK–RAS pathway is probably one factor that accounts for increased RFC mRNA expression in urothelial carcinomas. In non-urothelial carcinomas, however, RAS mutations are infrequent and other mutations come into play (p16 & p15 deletion [31], [32], Bax, Bak & EGFR overexpression and p53 inactivation [32]). Hence, we expect different genetic events to have led to the modest increase in RFC mRNA level in non-urothelial carcinomas, and possibly different TFs involved in RFC transcription activation. This is further supported by the fact that increased RFC expression is very likely to be associated with inactivation of tumor suppressor genes, where 5-Methyl tetrahydrofolate (5-CH3-THF) is a cofactor in the de novo synthesis of S-adenosylmethionine that participates in methylation of CpG islands. RFC is the main entry route for THF and increased RFC expression would accordingly lead to accumulation of the methylated reduced folate [11], [15] which may eventually lead to the inactivation of various tumor suppressor genes like; p16, p15 and/or p53 [34], [35].

Further, based on our present findings, RFC overexpression implies that urothelial bladder carcinomas would be intrinsically more sensitive to antifolates whereas non-urothelial carcinomas would show a less favorable response to antifolate chemotherapy. This speculation may explain why the MVAC regimen exhibits only limited antitumor activity against non-transitional cell cancer while response rates up to ∼70% were reported in TCC in the same study [9].

Our findings showing that RFC mRNA increased expression is not associated with tumor grade or stage implicate that increased expression of RFC mRNA seems to happen early in the process of multi-step tumor progression. This hypothesis is further supported by consistent RFC mRNA expression increase among the different age groups in our study. However, it has been reported that only ∼15% of the low-grade superficial tumors will proceed to infiltrate the musculature and that high-grade muscle-invasive tumors, either originate from flat carcinoma in situ (CIS)/severe dysplasia or arise *de novo* [29]. Accordingly, an alternative hypothesis could be that increased RFC mRNA expression occurs as a consequence of independent events among the different types of bladder carcinomas. Interestingly, all non-urothelial tumor biopsies utilized in this study were muscle-invasive pointing to the unfavorable diagnostic features of non-urothelial tumors as was already suggested by Erdemir *et al.* [19]. The clinical significance of squamous differentiation seems to be an unfavorable prognostic feature due to its association with high grade invasive tumors.

Although a significant difference was detected between RFC expression levels in bilharzial- and non-bilharzial-associated bladder cancers, this finding cannot be conclusive since only 7 cases (18% of total) of bilharzial-associated bladder cancer were included in this study (c.f. 32 non-bilharzial-associated case). Further, the higher level of RFC mRNA expression in BA-TCC (2 specimens) *versus* the SCC or mixed types (5 specimens) may also imply that RFC increased mRNA expression is actually independent from bilharzial infection.

Worth mentioning (since this supports the relevance) is that the characteristics of the bladder cancer specimens utilized in this study were representative of bladder cancer patients' population with regards to gender, age and histological types. The ratio of female∶male patients was ∼1∶3. The mean age of all bladder cancer patients included in the study was 60, where the mean age of the non-urothelial groups was lower than that of the urothelial bladder carcinoma patients (53 *vs.* 60) indicating the aggressive nature of the non-urothelial variants of bladder cancer. In addition, 64% of the patients had urothelial carcinomas, whereas only 24% presented with the non-urothelial variants. These demographic data were in accordance with the changing pattern of bladder cancer in Egypt previously reported by Felix *et al.* in 2008 [5], thus, highlighting the emergence and prevalence of the transitional cell type and retraction of the non-urothelial types.

**Figure 2:**
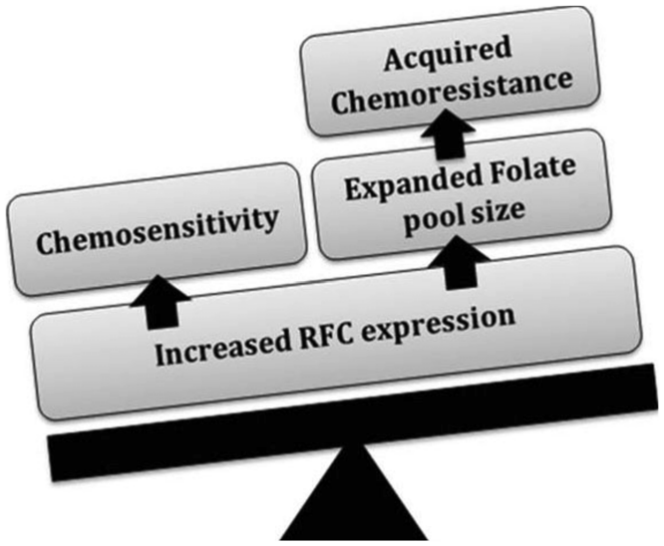
RFC is a double-edged weapon. A diagram depicting the proposed double-edged role of RFC in cancer chemotherapy. Increased expression is predictive of antifolates (MTX) transport. At the same time, increased expression of RFC will lead to folate pool size expansion forcing a negative feedback inhibition on the uptake of antifolates.

Conclusively, RFC expression can be considered as a double-edged weapon (Figure 2). Increased RFC expression would promote chemosensitivity to antifolates while, expanding intracellular folate pool size; which may lead to acquired resistance. This notion is further supported by the finding that loss of folate exporters led to resistance to antifolates through an expansion of the folate pool size [21]. Particularly, in bladder carcinomas, Rady *et al.*, (unpublished results) reported that BCRP (exports mono-, di-, and triglutamates of folates) expression was decreased, whereas MRP3, which have restricted ability to export only monoglutamate folates, was found to be overexpressed [Unpublished work by Rady et al. 2009]. Moreover, MRP3 was significantly higher in the urothelial relative to the non-urothelial bladder carcinoma variant [Unpublished work by Rady et al. 2009]. These variations in the expression levels of antifolates efflux and influx pumps imply that the utilization of a panel of genetic biomarkers for each tumor type may be more appropriate for defining response to chemotherapy rather than a single independent genetic marker.

### Conclusions and Future Directions

The present study was undertaken to investigate alterations in RFC expression among the different variants of bladder carcinomas. RFC mRNA expression was shown to be type-dependent but stage- and grade-independent. Although differentiation between urothelial and non-urothelial bladder carcinomas can be done by microscopic examination, our ultimate goal would be to integrate molecular diagnostics into clinical practice. Prospective evaluation of a panel of folate transporters (like; RFC, BCRP, and MRP3), can predict response to antifolate therapy, suggest new therapeutic strategies and hence, improve the treatment outcome for individual bladder cancer patients. The differences described between urothelial and non-urothelial carcinomas of the bladder could reflect different etiologies and/or risk factors. A larger study designed to provide correlations of the extent of RFC methylation with clinicopathological characteristics such as age, sex, histological grade, stage, and progression could provide additional useful information about the etiology of RFC altered levels of expression among different types of bladder cancers. Other known DNA methylation targets in bladder might also be included to see the effect of higher expression of RFC and higher folate transport on DNA methylation.

## Supporting Information

Figure S1 **RNA Quality Assessment.** Agarose Gel Electrophoresis of total RNA minipreps. Specimens' lanes are labeled according to their code number assigned to them after collection. Observing sharp bands of the 28S rRNA (4.8 kb) and 18S rRNA (1.8 kb) was the indication for intact RNA (T82, T65, S22 and T38). S22 and S38 contain traces of genomic DNA, however the RFC and β-actin primers were designed to span exon/intron junction to minimize genomic DNA amplification. T118 showed partial degradation but the 28S and 18S bands were very faint; the total RNA yield of T118 was only 2 µg. T107 showed degraded RNA and was excluded from further analysis. RNA (arrows) and DNA size markers (1031-80 bp) were included for comparison. This image was photographed using UVIsoft image acquisition system (Tokyo, Japan).
(TIF)

Figure S2 **Melting Curve Analyses.** Melting curve analysis for demonstrating the primers' specificity. Panel A: Dye fluorescence drops rapidly when the DNA melts. The melting point is defined as the inflection point of the melting curve, which is easiest determined as the maximum in the negative 1st derivative of the melting curve. The dissociation curves for both the target (RFC) and reference (ACTB) genes are shown. Each product displays a single sharp peak indicating the specificity of the primer pairs and absence of primer-dimers or non-specific amplification products. NTC either didn't record a Ct value or recorded Ct values in the range between (35–38) which differ by more than 5cycles from the highest Ct value recorded by the samples (not shown). The Flat curves of the ROX passive dye indicate no spiking (not shown). Panel B: Agarose gel electrophoresis of conventional PCR product using reverse transcribed cDNA of one tumor sample (T42) and reverse transcribed cDNA of the calibrator (commercially available, normal bladder RNA, Clonetech). Single bands of the correct sizes for RFC and the reference gene indicate the specificity of the products.
(TIF)

Table S1 **Clinical characteristics of bladder cancer patients.**
(XLS)
