# Supplementary figures and images for: Expression of RFC/SLC19A1 is Associated with Tumor Type in Bladder Cancer Patients
Source: PLoS One. 2011 Jul 8;6(7):e21820. doi: 10.1371/journal.pone.0021820 (PMC3132752; doi:10.1371/journal.pone.0021820)

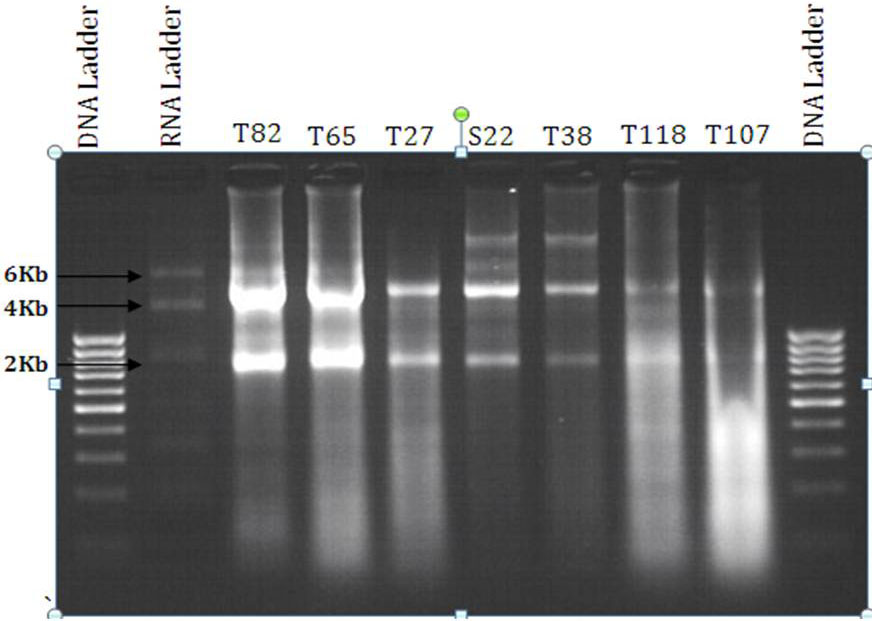

Supplement: Figure S1 — RNA Quality Assessment. Agarose Gel Electrophoresis of total RNA minipreps. Specimens' lanes are labeled according to their code number assigned to them after collection. Observing sharp bands of the 28S rRNA (4.8 kb) and 18S rRNA (1.8 kb) was the indication for intact RNA (T82, T65, S22 and T38). S22 and S38 contain traces of genomic DNA, however the RFC and β-actin primers were designed to span exon/intron junction to minimize genomic DNA amplification. T118 showed partial degradation but the 28S and 18S bands were very faint; the total RNA yield of T118 was only 2 µg. T107 showed degraded RNA and was excluded from further analysis. RNA (arrows) and DNA size markers (1031-80 bp) were included for comparison. This image was photographed using UVIsoft image acquisition system (Tokyo, Japan). (TIF) [file pone.0021820.s001.tif]

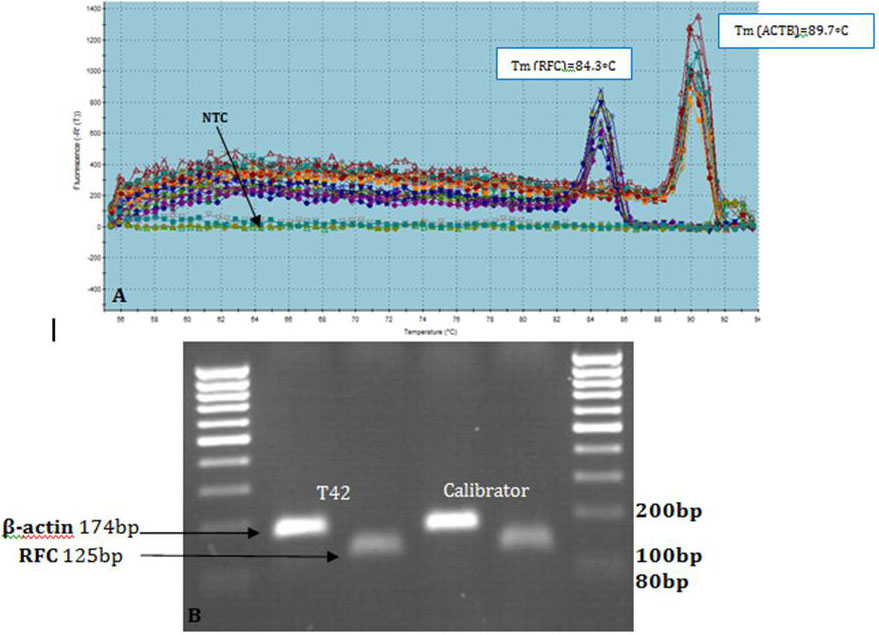

Supplement: Figure S2 — Melting Curve Analyses. Melting curve analysis for demonstrating the primers' specificity. Panel A: Dye fluorescence drops rapidly when the DNA melts. The melting point is defined as the inflection point of the melting curve, which is easiest determined as the maximum in the negative 1st derivative of the melting curve. The dissociation curves for both the target (RFC) and reference (ACTB) genes are shown. Each product displays a single sharp peak indicating the specificity of the primer pairs and absence of primer-dimers or non-specific amplification products. NTC either didn't record a Ct value or recorded Ct values in the range between (35–38) which differ by more than 5cycles from the highest Ct value recorded by the samples (not shown). The Flat curves of the ROX passive dye indicate no spiking (not shown). Panel B: Agarose gel electrophoresis of conventional PCR product using reverse transcribed cDNA of one tumor sample (T42) and reverse transcribed cDNA of the calibrator (commercially available, normal bladder RNA, Clonetech). Single bands of the correct sizes for RFC and the reference gene indicate the specificity of the products. (TIF) [file pone.0021820.s002.tif]
